# Insights from the development of an online toolkit to promote community engaged substance use research in the Southwest Borderlands Region

**DOI:** 10.21203/rs.3.rs-6985543/v1

**Published:** 2025-09-01

**Authors:** Andrew Gorvetzian, Maria Sanchez, Christina Phillips, Joseph Allbright, Alexandra Yonkovig, Cynthia Killough, Randall Benally, Janet Page-Reeves, Mary Alice Scott, Kimberly Page, Ivan de la Rosa

**Affiliations:** University of New Mexico; University of New Mexico; University of New Mexico; New Mexico State University; New Mexico State University; University of New Mexico; University of New Mexico; University of New Mexico; University of North Carolina-Chapel Hill; University of New Mexico; New Mexico State University

**Keywords:** Community engagement, substance use research, participant diversity in clinical trials, borderlands region, interdisciplinary scholarship

## Abstract

The Exploring Health Beliefs for Community Engagement and Diversity in Clinical Trials (EXPLORE) Toolkit is an online resource designed to support researchers and frontline workers addressing substance use in the Borderlands region of the Southwestern United States. Developed by an interdisciplinary team from the University of New Mexico and New Mexico State University, the toolkit aims to enhance engagement of underrepresented communities in substance use clinical trials through culturally sensitive, community-centered research practices. This paper presents the design, creation, and testing of the toolkit through multiple phases from initial design to final evaluation. Quantitative findings showed strong endorsement of the toolkit, with 79% of participants rating it “Very important” for promoting culturally competent research. Domain-specific ratings averaged 4.48–4.61 on a 5-point scale, with highest ratings for “Building on Community Strengths” (4.61). Post-testing showed improved attitudes toward clinical research participation (pre: 7.19, post: 7.94 on a 10-point scale) and enhanced understanding of research terminology (pre: 7.97, post: 8.74). Qualitative findings revealed critical themes including the importance of cultural appropriateness, community gatekeepers’ roles, and confidentiality concerns, particularly in rural and border communities. The EXPLORE Toolkit demonstrates promise for bridging research-community gaps while highlighting the value of interdisciplinary, community-centered approaches. Results suggest its potential for enhancing diverse community participation in clinical trials while emphasizing the need for ongoing adaptation to specific community contexts. The toolkit is available at the following link: https://sites.google.com/view/exploretoolkitcopy/home?authuser=0

## INTRODUCTION

The Exploring Health Beliefs for Community Engagement and Diversity in Clinical Trials (EXPLORE) Toolkit is an online resource developed to support researchers and frontline workers addressing substance use in the Borderlands region of the Southwestern United States and beyond. The region is characterized by high rurality, deeply established and underrepresented Hispanic and American Indian communities, and substantial migrant Hispanic and Latino populations. These states face a devastating substance use disorder (SUD) crisis, further exacerbated by pervasive health disparities, creating an urgent need for innovative, rigorously evaluated solutions. (Palau et al. 2024; Weinand et al. 2019; Segel and Winkelman 2021) Although some tools for increasing engagement in research exist, no compendium of tools aimed at advancing inclusion and engagement of diverse and minority groups in clinical research uses a conceptual approach that incorporates health beliefs, research literacy, and vulnerability. To fill this gap, an interdisciplinary team of epidemiologists, social workers, anthropologists, and public health professionals from the University of New Mexico and New Mexico State University designed the EXPLORE Toolkit to enhance engagement with underrepresented communities in substance use clinical trials. The Toolkit employs a strengths-based approach to community engagement, addressing equity, stigma, and participation barriers, while promoting culturally sensitive and community-centered research practices. It serves as a valuable resource for clinical trials researchers, community health workers, and other frontline workers seeking to engage underrepresented populations in clinical trials research throughout Southern New Mexico and beyond, while reflecting insights from interdisciplinary, community-engaged scholarship.

### Interdisciplinary approaches to community engaged research:

The fields of public health, epidemiology, and clinical trials research demonstrate growing commitment to meaningful community engagement.[1–4] Contemporary calls for interdisciplinary approaches to complex issues have fostered collaborations among researchers from many disciplines, including social work, psychology, and social and medical anthropology.[5, 6] This integration of disciplinary perspectives offers promising pathways for improving inclusion and engagement of diverse populations in clinical research while enhancing the relevance and translation of research findings into practice.

Underrepresentation in clinical trials research stems from multiple factors: limited knowledge about clinical research, mistrust of researchers, inadequate healthcare access, geographic constraints, and socioeconomic factors including poverty and discrimination based on race, gender, and/or sexual orientation.[1, 2, 7–13] This underrepresentation impacts both communities and researchers. Communities often lack understanding of research processes and potential benefits, while providers and community health groups may have insufficient knowledge about clinical research and its alignment with community priorities. Prior to the development of this Toolkit, no comprehensive resource existed which specifically aimed at advancing inclusion and engagement of diverse and minority populations in clinical research.

Before delving into the development of the Toolkit, it is important to define what is meant by “community”. “Communities” can be defined in many ways. The U.S Centers for Disease Control and Prevention (CDC) broadly defines it as a “geographic area, a group of people with shared interests, or a feeling of teamwork and fellowship”. [14] In conversations about equity in health research, the concept of “community” refers to non-academic partners, participants, and other interested parties with an emphasis on inclusivity. However, it is important to recognize that individuals belong to many different, overlapping, fluid, and sometimes contested communities. This complexity is often even more pronounced among or within minoritized communities. Therefore, when considering “community engagement,” it is important to have a concrete and shared understanding of what is meant by “community” rather than relying on aspirational assumptions, and consideration should consider the context of specific research projects. Early in the research process investigators should engage collaborators to define important considerations, for example data ownership and sharing parameters, — and establish what these concepts should look like in their specific project. The resulting project data sharing framework should follow and honor the outcomes of this collaborative process.

While the concept of community and methods of community engagement are multifaceted and diverse, significant gaps exist in scientific research, particularly in substance use clinical trials. The EXPLORE Toolkit represents one solution to addressing these gaps, providing a structured framework for meaningful community-engagement while acknowledging the inherent complexity of community-based research.

## METHODS

### Toolkit Design and Testing

#### Toolkit Design

Development of the toolkit began in September of 2021 when the team convened. The three-year development process unfolded through several distinct phases, as outlined below:

1. Review and evaluation of related toolkits and sources
2. Development of EXPLORE domains
3. Design of online toolkit and platform
4. Focus groups for beta version of toolkit
5. Toolkit revisions
6. Toolkit theater testing
7. Final toolkit revisions

#### Review and evaluation of related toolkits and sources:

During the initial phase, the team conducted a comprehensive review of existing toolkits to identify gaps and determine the unique contribution the EXPLORE Toolkit could make. The search encompassed resources focused on public health, community engagement, patient centered outcomes and care, health beliefs, and strategies for engaging minoritized and underrepresented communities in research. Through this broad review, the team identified 17 existing tools, toolkits, and frameworks that met search criteria. Each resource underwent systematic analysis based on several key elements: mission and stated goals, components, engagement methods and principles, adaptable tools, user experience and design principles, and limitations (see Table 1). Following the initial evaluation of existing toolkits, our team considered how these resources could inform both the content and design of the EXPLORE Toolkit. As discussed below, our toolkit directly incorporates many of the tools and resources identified in our initial search. Rather than duplicating existing work, we aimed to create a comprehensive “one-stop shop” that seamlessly integrates previously developed tools with our new contributions.

#### Development of EXPLORE domains:

The development process then focused on establishing a framework to guide the Toolkit’s design. This framework serves dual purposes: providing researchers with readily accessible tools for project design and execution, while also functioning as an educational resource. The Toolkit helps researchers, health workers, and community members to understand not only research processes but also the broader socioeconomic, historical, and political factors that shape research engagement. Through an iterative process, the team organized the Toolkit into five distinct domains:

Domain 1: Preparing Researchers to Go into Communities

Domain 2: Structural Factors that Influence Participation

Domain 3: Building on Strengths to Support Participation

Domain 4: Engagement and Communication Methods

Domain 5: Tools and Resources

**Domain 1** serves as an introduction to the Toolkit, establishing its purpose and target audience. The Toolkit addresses the needs of three primary user groups: 1. experienced researchers seeking to enhance diversity and inclusion among participants, 2. clinicians and professionals involved in recruitment or referral for clinical trials, and 3. community health workers who interface directly with community members regarding research participation.

**Domain 2**, “Structural Forms of Discrimination,” presents five interconnected thematic areas: 1 - Geography; 2 - Racism, skepticism, and distrust of researchers; 3 - Economic and material factors; 4 - Education, stigma, and health literacy; and 5 - Gender and sexuality. These themes form a comprehensive framework illustrating how various factors intersect to impact clinical trial participation. The framework examines how social and economic policies, healthcare resource distribution, and social stratification based on multiple identities (race, ethnicity, religious affiliation, immigration status, ability, gender identity, and sexual orientation) create complex barriers to participation among marginalized populations. Each theme is directly linked to specific challenges in recruitment and retention for clinical trials.

**Domain 3** presents a strengths-based approach to community engagement, functioning in parallel with the structural discrimination framework outlined in Domain 2. Building upon the five previously identified themes, this domain offers a framework for reframing perceived barriers as community assets. The domain provides practical strategies for researchers and health workers to leverage these strengths when designing and implementing clinical trials and other interventions. An example of this framework’s application is presented below.

**Domain 4**, Communication and Engagement Strategies, features two illustrative vignettes depicting hypothetical researcher-community interactions. These scenarios highlight the critical role of language and context in community engagement. The vignettes demonstrate both the importance of using plain language when explaining research concepts and the necessity of understanding social dynamics within specific communities.

**Domain 5** presents core components and provides users with comprehensive resources for practical implementation. This section features extensive tables of tools organized by both Domain and Theme, enabling efficient navigation based on specific needs. The resources include links to existing toolkits, original tools developed by our team, and curated external resources. These tools support various aspects of community engagement, from facilitating focus groups and building community trust to providing educational materials about clinical trials and offering evaluation frameworks for pre- and post- trial phases. Additionally, the section includes specific tools addressing the communication challenges highlighted in Domain 4.

### Toolkit Testing

#### Focus groups

Following initial Toolkit development, the team initiated user testing to evaluate experience, perceived usefulness, and content through recruitment of potential users and/or communities in the target region. We considered the geographic area, initially as Southern New Mexico, and reached out to groups in healthcare including from clinics, organized community groups (for example the Dona Ana Wellness Institute), community health workers, and researchers linked to academic groups. Not unexpectedly, as clinical trial research is not common in this region, efforts to recruit clinical researchers were not optimal. And as the COVID-19 pandemic continued, recruitment for focus groups was challenging. The team expanded their recruitment strategy to include a broader pool of potential users, and we shifted to single-person interviews by videoconference (n = 10). The result was inclusion of some nursing professionals, frontline providers including Emergency Medical Technicians, and community health workers. These sessions did yield valuable insights regarding user experience and suggested that the Toolkit’s utility could be extended beyond its originally intended audience.

#### Theater Testing

Following focus group feedback incorporation, the team conducted theater testing to assess usability, content, and relevance. In health intervention design, theater testing evaluates the feasibility, acceptability, and potential effectiveness of an intervention or research approach before it is fully implemented.[15, 16] This method involves a metaphorical “staging” of the research process rather than theatrical performance. The team identified CTN-107 (NCT05123027) through the NIDA Clinical Trials Network as a prototype study for Toolkit evaluation, as it was feasible that this study could be done in southern New Mexico. The study assessed the deployment of a peer intervention linking opioid overdose survivors to treatment for opioid use disorder (OUD).[17] The team partnered with the New Mexico Department of Health (NMDOH), Office of Community Health Workers (OCHW), focusing on Southern New Mexico. Community Health Workers (CHW) were selected for testing due to their integral role in New Mexican communities and their position as trusted facilitators and gatekeepers.[18, 19]

Building on previously established relationships, the Community Engagement Liaison Specialist at the Clinical and Translational Science Center facilitated connections with Promotoras de Salud (“*Promotoras*”; CHWs working with Latino/a/x communities), enabling the successful recruitment of participants for the initial Theater Test. The initial theater test included 60 promotoras in rural southern New Mexico. CHW participants were recruited during a scheduled promotoras meeting in January 2024, held in a “*colonia”* (rural unincorporated settlement) in Doña Ana County. [20, 21] Written informed consent was waived by the UNM HSC IRB, as theater testing presented minimal risk and all data were collected anonymously. Information sheets in English and Spanish detailing procedures, risks and benefits were provided to all participants. Two Spanish-speaking investigators (Kim Page and Ivan de la Rosa) facilitated the Theater Testing event, which was conducted primarily in Spanish with simultaneous English translation for the small minority (< 6) of non-Spanish speakers. Participants received a $50 merchandise card, and completed pre- and post-test surveys assessing feasibility, acceptability, community interest, and research literacy (with questions about understanding the terms *randomization, consent form, protocol*, and *institutional review board)*. The quantitative survey items were created for the purposes of this project and were not adapted from another source.

#### Theater Testing Activities

The session began with facilitators introducing the SW CTN EXPLORE project and its goals of enhancing diverse community representation in substance use research. The facilitators introduced themselves and the team, and presented a brief overview of the process for the theater test. Participants received bilingual Toolkit copies and electronic access via QR code. The team then staged a “request to consider research” intervention, presenting CTN-107’s study components including informed consent review and implementation procedures. Participants were randomly assigned to four breakout groups, each focusing on a specific Toolkit domain. Groups analyzed vignettes aligned with the intervention using their assigned domain’s tools. For example, the Domain 4 group evaluated communication strategies through hypothetical researcher-community member interactions. Following breakout sessions, groups reconvened to share findings and synthesize feedback.

Pre-and post-evaluations were administered to theater testing participants to assess attitudes and perceptions towards the toolkit, and which included closed and open-ended questions. Closed questions included 10-point scales asking participants to rate feasibility, acceptability, community interest level, and research literacy. For example: “On a scale of 1 to 10, how likely is it that a clinical research study could run successfully in your community, with 1 being “not at all likely,’ to 10, “highly likely.” Additionally, following toolkit testing, participants were asked to provide feedback on their impressions of the toolkit using a 5-point scale to rate: usability, cultural relevance, dialogue facilitation, belief that the toolkit provides valuable information for researchers, and likelihood of recommending it to others. Finally, participants were asked to rate the importance of the domain topics. We did not perform statistical tests comparing pre- and post-theater testing responses due to the high baseline scores observed pre-theater testing, which likely reflect social desirability bias rather than true baseline knowledge, making statistical comparisons inappropriate and potentially misleading.

Following this first theater test, the NMDOH Office of Community Health Worker Director invited the team to conduct a second theater testing event with CHWs serving American Indian communities in Northern New Mexico. In this second event, participation was limited to 25 CHWs for enhanced engagement. The second theater test maintained the core methodology while incorporating minor refinements to enhance data collection: 1) Participants used post-it notes to record their thoughts before verbal group discussions; 2) smaller group sizes enabled facilitators to gather more detailed individual feedback; 3) the session was conducted in English. These modifications aimed to optimize participant engagement and data quality while maintaining the testing protocol’s fundamental structure. The same pre- and post-evaluation tools were used for this session.

The EXPLORE team subsequently conducted a comprehensive review or “debrief” of procedures, participant feedback, and survey data to inform Toolkit refinements and future theater testing modifications.

##### Ripple Effects Mapping:

Ripple Effects Mapping (REM), an innovative participatory evaluation technique was used to assess the EXPLORE Toolkit development process.[22–24] REM illuminates interconnections between program elements, activities and impacts, creating visual fractal “maps” that illustrate change pathways. Using XMind 8 Pro Mind Mapping Software, the team documented complex processes and captured unforeseen impacts that traditional evaluation methods might miss. In July 2023, the team conducted a retrospective evaluation discussion to co-create a visual REM “map” of the data, guided by four key questions: 1. What did we learn through Toolkit creation? 2. What occurred during the development process? 3. How did this project impact participants? 4. How did this project influence our research approaches?

## RESULTS

### Quantitative results:

Domain-specific feedback revealed varying perspectives across the four main areas. For Domain 1 (Preparing Researchers), participants emphasized the need for community-specific approaches while acknowledging the clear instructions provided. Domain 2 (Structural Discrimination) resonated strongly, with participants noting its relevance to New Mexico’s changing demographic landscape. Domain 3 (Building on Strengths) received mixed feedback, with some seeing it as empowering while others questioned its universal applicability across New Mexico communities. Domain 4 (Engagement Methods) was particularly valued for its practical communication strategies and emphasis on resource awareness.

Evaluation of the EXPLORE Toolkit revealed strong positive participant responses across multiple dimensions. The Toolkit’s *usability* received the highest rating (M = 4.26, SD = 0.89 (on a 5-point scale)), followed by its ability to promote dialogue about substance use disorders (M = 4.10, SD = 1.18), and cultural relevance (M = 4.07, SD = 1.11) (Table 4). Regarding recommendations, 64% indicated they were “very likely” to recommend the Toolkit to others, with an additional 15% “likely” to recommend it. Only 11% remained neutral, and a minimal percentage (6%) were unlikely to recommend. All of the Toolkit domain topics received strong endorsement (very important) with mean scores ranging from 4.48 (domain topic 1), to 4.61 (domain topic 3). A majority (79%) of participants found the Toolkit’s guidance to be valuable for researchers working with communities, while the remaining 21% found it somewhat valuable. No participants rated it as lacking value.

**Figure 1.**
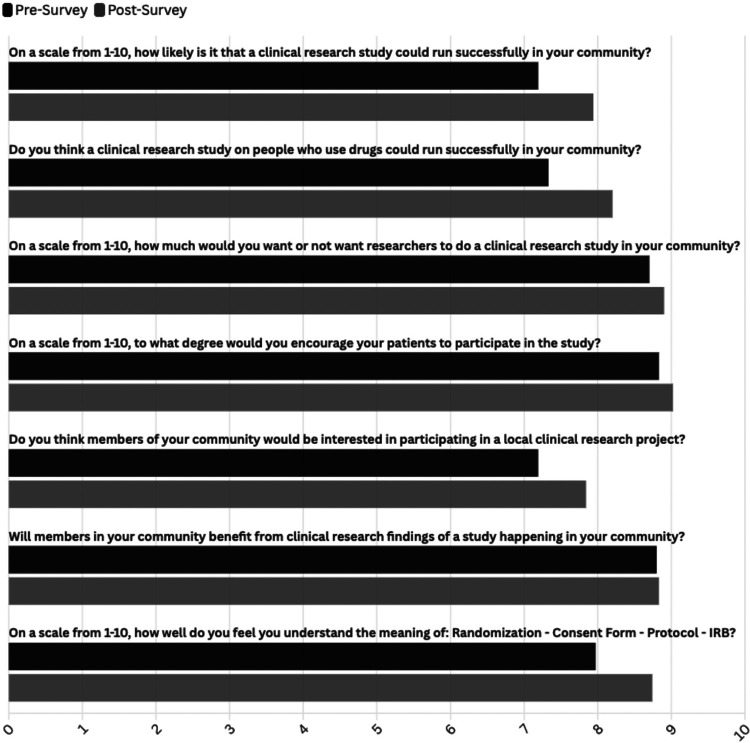
Attitudes and Perceptions towards Clinical Research in the Community

When the participants were asked how likely a clinical research study could run successfully in their community, the post-survey rating (M = 7.94, SD = 2.09) was higher than the pre-survey rating (M = 7.19, SD = 2.42). When the participants were asked if they think clinical research on people who use drugs could run successfully in their community, the post-survey rating (M = 8.20, SD = 1.95) was significantly higher than the pre-survey rating (M = 7.33, SD = 2.34). Participants were asked how much they would or would not want researchers to do a clinical research study in their community, the post-survey rating (M = 8.90, SD = 1.63) was slightly higher than the pre-survey rating (M = 8.70, 2.02). When asked to what degree the participant would encourage their patients to participate in the study, the post-survey rating (M = 9.02, SD = 1.65) was also slightly higher than the pre-survey rating (M = 8.83, SD = 1.77). Participants were asked if they think members of their community would be interested in participating in local clinical research projects, the post-survey rating (M = 7.84, SD = 2.15) was higher than the pre-survey rating (M = 7.19, SD = 2.25). Participants were asked to rate their opinion about how members of their community could benefit from clinical research findings from the clinical study being done in their community, the post-survey rating (M = 8.83, SD = 1.55) was slightly lower than the pre-survey rating (M = 8.80, SD = 1.60). Lastly, participants were asked if they understand the meaning of the terms: randomization, informed consent, form, protocol, and institutional review board (IRB), the post-survey rating (M = 8.74, SD = 1.83) was significantly higher than the pre-survey rating (M = 7.97, SD = 2.45). The differences between the post-survey and pre-survey ratings show that participant attitudes and perceptions about the toolkit improved after learning more about the toolkit during the facilitator-led theater testing sessions (see Fig. 1).

The Toolkit demonstrated adaptability across different geographic regions and cultural backgrounds, as the results showed the Toolkit to be constructive both for community health workers serving Hispanic communities in Southern New Mexico and American Indian communities in Northern New Mexico.

### Qualitative results:

Thematic analysis revealed three primary themes: toolkit effectiveness, cultural appropriateness and community connection, and domain topic relevance. Within these, several sub-themes emerged, including community respect, cultural sensitivity, community involvement, and confidentiality. 78 total participants participated in producing these results.

Toolkit effectiveness and usability: Participants consistently emphasized the Toolkit’s practical utility, particularly noting Domain 1’s effectiveness in preparing researchers for community engagement. As one participant noted, “*This topic was explicit in its instructions, and it was clear to me what the researchers were explaining*.” The small group format proved particularly valuable, with participants appreciating “*having facilitators in our group*” to guide domain exploration. However, participants offered crucial feedback for improvement, particularly regarding accessibility. “*Use simpler language*” and consider a “*lower reading level*” were common suggestions. Several participants recommended enhancing visual presentation, requesting “graphics or pictures” to reduce written information and “bigger font for accessibility.” Time management emerged as a critical consideration, with participants emphasizing the need for “more time on the topics” to ensure effective collaboration.

Cultural Appropriateness and Community Connection: Cultural competency emerged as fundamental to successful engagement and implementation. As one participant directly stated, researchers must “*know the culture that you are doing the study in*.” Another emphasized that researchers “*must be culturally competent and understand why community members may be hesitant to work with researchers*.” Community involvement emerged as crucial, with distinct regional perspectives. Northern New Mexico participants emphasized elder involvement, while Southern New Mexico participants highlighted community health workers’ role. As one participant noted, “*If you are going to carry out this project, offer help and respect the person that asks for help and have follow-ups with the person*.” Confidentiality concerns were particularly salient in rural communities. One participant observed, “*Making sure the community knows everything is confidential. Having worked with rural communities before, [many] are reluctant due to immigration status*.”

Domain topic relevance: The theater testing sessions yielded comprehensive feedback across all four domains, revealing both strength and areas for refinement in each section of the toolkit.

*Domain 1: Preparing Researchers*. The feedback on researcher preparation revealed important tensions between standardization and community-specific approaches. While some participants found the domain generalizable, others emphasized its limitations, noting “*Minimal. Each community is unique*.” This contrast highlighted a crucial insight: while basic principles of community engagement can be standardized, successful implementation requires flexibility and cultural competency. Community members may be more hesitant to work with researchers who are unwilling to adapt to the specific characteristics of the particular community with which they are working. This feedback underscored the importance of building trust through understanding community-specific contexts and historical experiences with research.

*Domain 2: Structural Forms of Discrimination*: This domain generated particularly robust discussion about systemic barriers and community impact. Participants noted its “*very high impact*” and emphasized that “*to address it, you need to see the problem*.” The domain’s relevance to New Mexico’s dynamic demographic landscape emerged clearly, with one participant observing, “*The landscape of NM is constantly changing, and I am not always sure how to formulate that, but this was a strong process on non-discrimination*.” Participants particularly valued the domain’s emphasis on respect and non-judgment, with several noting how it “*helps us remember the importance to not judge anyone*.” The focus on inclusion was seen as both impactful and effective in addressing structural discrimination, with participants emphasizing how this approach could help break down traditional barriers to research participation.

*Domain 3: Building on Strengths*: The evaluation of this domain revealed its potential as a catalyst for community development. Participants saw it as “*a door to increase services in the community*” and praised its relevance to current community needs. However, some participants noted important limitations, with one observing that “*It met the needs of some areas, but I struggled to see it come together for all communities here in NM*.” This feedback highlighted the challenge of creating resources that resonate across New Mexico’s diverse communities. The domain’s emphasis on community empowerment emerged as particularly valuable, with participants noting how it effectively “*draws attention to the problems or challenges happening in the community*” while maintaining a strengths-based approach. This balance between acknowledging challenges and building on existing community assets was seen as crucial for meaningful engagement.

*Domain 4: Engagement and Communication Methods*: The final domain received strong endorsement for its practical utility and potential for immediate application. Participants found multiple aspects of this domain effective, noting how “*All of the topics that involve the community help us*” and emphasizing that “*Communication is the important aspect to get the message out there for results*.” The domain’s approach to building community confidence was particularly well-received, with participants appreciating its potential to empower community members. A key insight emerged regarding resource awareness, with one participant emphasizing that “*If you let the community know about the resources or help that is given, the community will respond*.” This feedback highlighted the crucial connection between effective communication, resource awareness, and community engagement. Participants also valued the domain’s practical strategies for maintaining ongoing community dialogue and building sustainable research relationships.

#### Ripple Effects Mapping Results:

The REM process revealed significant insights about both the toolkit’s development process and its potential for transforming research practices. Three interconnected spheres of impact emerged: shifts in researcher perspectives, enhancement of community-research relationships, and institutional transformation.

Shifts in Researcher Perspectives: Through the REM process, team members reported profound changes in their understanding of community engagement. Researchers noted how their initial assumptions about “efficient” research processes were challenged and transformed. As one team member reflected, “*What we thought would be a straightforward process of creating guidelines became a journey of understanding how communities view research differently*.” The process highlighted how structural violence impacts individuals affected by substance use disorder, revealing intersections that weren’t initially apparent to the research team. The team’s appreciation for local knowledge deepened significantly. Rather than viewing community input as simply informative, researchers came to more fully appreciate it as fundamental to successful research design. This shift manifested in practical changes, such as developing alternative methods for presenting academic information and creating more accessible formats for sharing research processes. As one researcher noted, “*We had to unlearn our academic training to really hear what communities were telling us*.”

Enhancement of Community-Research Relationships: The REM process highlighted the fundamental importance of trust-building and social relationships in community research. Team members identified specific moments where their understanding of community dynamics shifted, particularly regarding the role of informal networks and community gatekeepers. The process revealed how successful research requires moving beyond traditional academic approaches to embrace non-extractive, collaborative methodologies. The mapping exercise illuminated the critical role of humility in community-engaged research. Researchers reported gaining a deeper understanding of how their own biases and narrow perspectives could limit engagement. One team member observed, “*We realized that our ‘expertise’ sometimes created barriers rather than bridges to community understanding*.”

Institutional and Methodological Transformation: Perhaps most significantly, the REM process revealed how the toolkit development transformed understanding of clinical trial implementation, particularly in the context of community-based substance use disorder research. Key insights included: recognition that research benchmarks must be locally relevant rather than imported from different contexts; understanding that scale-appropriate research approaches may differ significantly from traditional clinical trial methods; appreciation for how regional differences affect implementation strategies; and recognition that success metrics may need redefinition in community contexts.

Challenges and Revelations: The REM process revealed important challenges that significantly informed toolkit development. Initial assumptions about recruitment were confronted when traditional approaches proved ineffective, forcing the team to reconsider fundamental aspects of community outreach. Concerns about confidentiality emerged, particularly in rural and border communities, highlighting the complex intersections of research ethics, trust, and community safety. These challenges catalyzed important innovations in approach. The team developed more flexible recruitment strategies that respected community rhythms and cultural practices, while creating enhanced confidentiality protocols that addressed specific community concerns. There was recognition of the need for longer relationship-building periods; and understanding that community timelines may differ from academic schedules.

## DISCUSSION

The EXPLORE Toolkit development process revealed several key insights about the complexities of community engagement in clinical trials research. What began as a targeted resource for researchers evolved into a versatile platform serving multiple stakeholders, from clinical researchers to community health workers and frontline responders. Particularly notable outcomes of this multimethod evaluation approach, combining focus groups, theater testing, and REM, yielded rich insights into both external engagement and internal team dynamics. Theater testing proved particularly valuable for assessing complex behavioral health interventions, revealing critical insights about social determinants of health, trauma, and the importance of cultural sensitivity. Findings emphasize that successful community engagement requires deep understanding of local cultural contexts and power dynamics.[1] The critical role of community gatekeepers emerged as fundamental to research success, requiring careful consideration of power relationships, negotiation of boundaries, and collaborative knowledge construction. This aligns with previous research highlighting gatekeepers’ importance in community-engaged research.[25–27] Cultural appropriateness proved essential for meaningful engagement. Participants consistently emphasized the need for locally relevant approaches, including incorporation of community testimonials and adaptation of materials for specific populations.[28] The particular needs of tribal communities emerged as a crucial consideration, highlighting the importance of cultural protocols and indigenous knowledge systems.[29] Multiple language availability and attention to confidentiality concerns, especially in rural and border communities where immigration status may affect participation, emerged as critical factors for success.

As the data shows, the EXPLORE Toolkit has potential to increase engagement for diverse populations in clinical trials research when used as a means of helping inform community members about all that clinical trials research entails. Nevertheless, trust-building and community empowerment are critical to successful engagement. The development process highlighted how successful research requires non-extractive collaborative approaches and emphasized the essential role of humility in community-engaged research. The toolkit’s effectiveness in empowering dialogue through education while maintaining accessibility for researchers addresses a critical need for resources that bridge the researcher-community divide. Furthermore, our rounds of theater testing in various communities in New Mexico demonstrated that the Toolkit has adaptability. That adaptation requires having effective cultural adaptation, humility, and respect.[30]

The cross-institutional collaboration between UNM, NMSU, and NIH demonstrated the value of diverse perspectives in addressing complex health challenges. The REM process revealed how interdisciplinary dialogue enhanced understanding of substance use disorder complexities in borderland communities. This collaborative approach led to innovations in presenting academic information in an accessible format while developing alternative methods for research interaction.

The REM process also revealed a crucial, often overlooked aspect of community-engaged research: the transformation of researchers themselves. Through REM, team members documented how their own perspectives and approaches evolved, challenging traditional academic paradigms about expertise and knowledge construction. As one researcher noted above, “*We had to unlearn our academic training to really hear what communities were telling us*.” This willingness to adapt and change proved fundamental to the toolkit’s development and success. The REM process highlighted how meaningful community engagement requires researchers to embrace vulnerability, acknowledge their own biases, and remain open to fundamental shifts in their understanding of research practices.[31] This finding suggests that effective community-engaged research tools must not only guide community interaction but also support researcher growth and adaptation. The EXPLORE Toolkit’s success thus depends not just on its content, but on researchers’ willingness to engage in this transformative process.

We note several limitations that warrant consideration. First, testing was conducted in simulated rather than realworld settings, potentially limiting understanding of practice implementation challenges. The geographic focus on the Southwestern United States may limit generalizability to other regions or communities. Initial testing focused primarily on CHWs rather than researchers. The long-term impact and sustainability of using a toolkit as designed here, require further evaluation. Finally, the toolkit’s effectiveness across different substance use disorder contexts needs further exploration.

### Future Directions

This paper marks the beginning rather than the conclusion of the EXPLORE Toolkit’s development. Future development should focus on real world implementation studies across diverse settings which will be crucial for understanding the toolkit’s practical utility and limitations. These studies should include both urban and rural contexts, and diverse cultural settings beyond the Southwest. Systematic evaluation frameworks need development to measure both immediate and long-term impact on research participation and community engagement. We are currently working on a dissemination plan, which includes adjusting some elements of the toolkit following feedback from a User Experience Evaluation (such as replacing the word “domain” for clarity, reformatting the reflection sheets, and adding QR codes to facilitate sharing the toolkit); targeted outreach via leveraging NIDA CTN Nodes and social media; and housing the toolkit on platforms like the NIDA Clinical Trials Network website. As dissemination progresses, we anticipate discovering additional applications and insights. The toolkit should establish clear pathways for ongoing adaptation and refinement, including creating formal feedback mechanisms for users to share experiences and suggestions, and developing protocols or regular content updates based on user experiences. Community review boards could help guide adaptations. Ensuring flexible frameworks remains a priority, however, to allow for cultural and contextual modifications. To ensure effective implementation, we included user guides, however, online training for different partner groups may be useful. Other technological improvements could include developing digital platforms for user networking, and accessible data visualization tools. All of these ideas emphasize the toolkit’s role as a living document that should grow and adapt through ongoing engagement with all users. Success will require commitment to collaborative development and responsive adaptation to emerging needs and insights.

### Conclusion

While the EXPLORE Toolkit does not claim to be a comprehensive solution to community engagement challenges in clinical trials research, it represents a significant step toward bridging research-community gaps. By facilitating meaningful dialogue and promoting community agency in healthcare research, the Toolkit contributes to the broader goal of ensuring more inclusive and effective substance use disorder research in the Borderlands and beyond. Its success demonstrates the value of interdisciplinary collaboration and community-centered approaches in addressing complex public health challenges.

## Figures and Tables

**Table 1:** Main Principles and Components of Existing Toolkits

Name of Toolkit	Mission/aim of Toolkit	Methods/Principles of Engagement	Components of Toolkit	Other useful info	Unique elements
ReachNet	The involvement of patients, caregivers, clinicians, and other healthcare stakeholders throughout the research process—from topic selection through design and conduct of research, to dissemination of results. Helping people make informed decisions to improve health care delivery and health outcomes.	Patients can contribute to and prioritize research topics; can participate beyond being subjects and more as consultants; be connected to the clinical research teams in meaningful ways	Script for medical assistant; Data use policy; Patient Consent forms	This is an upstream approach. Key is: who controls the narrative. How to include voices input for trials that are already designed.	Clinic champion (pg. 18): a role for how to integrate patients, providers, and other stakeholders
Brandeis Community Engagement Studio (CES) Toolkit	Providing an outline for an interactive, consultative model for community involvement in which community experts are seen as collaborators in research design, not subjects of studies	Key members of CES team: Community navigator (navigates community relationships with academic group); science navigator (works with investigators to facilitate communication with stakeholders); manager (works with navigators to ensure effective implementation); Facilitator for Studio testing, Researcher presenting research, and community experts (pgs. 12–18 for more details)	Facilitator CES guide; Tools for Recruiting Researcher; Researcher CES guide; Tools for recruiting community experts; Community Expert CES guide; Consent forms; CES evaluation survey		Good table on page 7 distinguishing between focus group and CES
Gathering of Native Americans (GONA) Toolkit	Complementing the guide above, this toolkit offers dozens of materials for team building, trust building, and engagement materials for facilitators working with Native american groups		Toolkits for Belonging, Mastery, Interdependence, Generosity and Healing from Intergenerational trauma; aslo one geared towards youth		Offers concrete examples of what centering “Indigenous ways of knowing” looks like in practice
Rain Barrel Communications Participatory Research Toolkit^1^	A toolkit with dozens of specific tools for participatory action research, with outlines of the tools, reflections from experiences of using the tools, and other insights. Tools have been used for myriad health issues across the world, including female genital mutilation, nutrition, AIDS, malaria, etc.	They can be used to conduct participatory situation assessments, monitor the extent to which interventions are being implemented according to plan or to measure effectiveness, i.e., changes in behavioral and social outcomes. The strength of these tools lies in the fact that they can be integrated directly into individual and social change communication programming, providing participants the ability and skills to create and analyze data. Many of the tools are specifically designed to build teamwork and to make research an enjoyable exercise. Additionally, these tools can be used to examine societal and cultural factors which are di cult to understand and decipher using traditional methods. They allow both participants and researchers to see the world in a different light. (direct citation from intro to document)	The toolkit is a list of all of the tools used. Description of tool, rationale, application, and tips for use. See document for all of the tools		Too many tools to look at, could be useful for teams to go through and see if any are useful. This is a good example of how to lay out tools, how to apply, and how to use the tools and what to expect.

[1] This toolkit, which was developed in coordination with USAID, is no longer accessible online. From their website: As of March 15 2025, The Communication Initiative (The CI) platform is operating at a reduced level, with no new content being posted to the global website and registration/login functions disabled. (La Iniciativa de Comunicación, or CILA, will keep running.) While many interactive functions are no longer available, The CI platform remains open for public use, with all content accessible and searchable until the end of 2025. Please note that some links within our knowledge summaries may be broken due to changes in external websites. The denial of access to the USAID website has, for instance, left many links broken. We can only hope that these valuable resources will be made available again soon. In the meantime, our summaries may help you by gleaning key insights from those resources (https://global.comminit.com/content/participatory-research-toolkit)

**Table 2:** Themes and subthemes explored in Domain 2: Structural Forms of Discrimination

Theme	Subtheme	Example
Geography	Transportation instability	Having limited access to transportation could make it challenging for an individual to make it to their appointments for clinical trials if they are conducted in person
Racism, skepticism, and distrust of researchers	Skepticism of the medical system	Medical providers often operate under outdated approaches with stigmatizing beliefs about substance use and substance use treatment which cause patients to feel judged and avoid healthcare systems.
Economic/material factors	Poverty	Not having enough money to live comfortably could impact an individual’s decision to participate in clinical research due to time constraints and perceived value for giving their time based on the compensation.
Education, Stigma, and Health Literacy	Health misinformation/literacy	Misinformation has been a growing concern for the scientific community with the widespread use of social media and news platforms, which are pervasive and often provide incorrect information. This prevalence could limit the trust that an individual has in the motivation for the trial and potential outcomes.
Gender and Sexuality	Gender Identity	Historically, female/woman-identifying individuals absorb much of the household labor and responsibility within a family unit, including childcare, which can limit the amount of time they are able to give to participate in clinical trials.

**Table 3:** Domain 3: Building on a Strengths-based to Support Participation

Barrier	In smaller communities it is more likely that people know each other or of each other. This can discourage participation in substance use research to avoid stigma and discrimination.
Strength	Video conferencing and other virtual communication resources have become more common in the wake of the COVID-19 pandemic. These tools facilitate more discrete participation in research.
Strategies	Virtual resources can reduce potential stigma against participating in clinical trial research as well as reduce barriers such as geographic distance from a research site. Virtual technology allows participants to be involved with privacy. Trials may be designed to reduce the amount of fact-to-face contact. There are a growing number of resources available for “Decentralized Clinical Trials” that may be well suited for rural areas. If your research takes place at a fixed site, consider how you can create a private entrance, or ‘backdoor’ into the site to maximize privacy and confidentiality.

**Table 4:** Toolkit Impressions and importance of toolkit domain topics

Impressions and views	Score	
	Mean	SD
Toolkit usability	4.26	0.89
Cultural Relevance	4.07	1.11
Promotes dialogue	4.10	1.18
Domain Topic Importance		
1: Preparing researchers	4.48	0.90
2. Structural discrimination	4.52	0.91
3. Building on strengths in the community	4.61	0.84
4. Engagement methods	4.52	0.90

## Data Availability

The datasets generated and/or analyzed during the current study are available in the Figshare repository, https://doi.org/10.6084/m9.figshare.29452202.v1
